# Gamma-glutamyl transferase and calculus of kidney incidence: a Mendelian randomization study

**DOI:** 10.1038/s41598-023-48610-7

**Published:** 2023-12-09

**Authors:** Peizhe Li, Yuewen Pang, Shuang He, Junyao Duan, Huijie Gong, Yongji Yan, Jing Shi

**Affiliations:** Department of Urology, Dongzhimen Hospital, Beijing University of Chinese Medicine, Hai Yun Cang On the 5th Zip, Dongcheng District, Beijing, 10000 China

**Keywords:** Genetics research, Genetics

## Abstract

Elevated Gamma-glutamyl transferase (GGT) levels are often suggestive of cholelithiasis, and previous studies have indicated that GGT is highly expressed in the urinary system. Therefore, we hypothesized that there may be an association between GGT levels and calculus of kidney (CK) incidence. To investigate this potential causal relationship, we employed Mendelian randomization (MR) analysis. Additionally, we analyzed the levels of other liver enzymes, including alanine transaminase (ALT) and alkaline phosphatase (ALP). The relationship between GGT levels and CK incidence was analyzed using two-sample Mendelian randomization. Summary Genome-Wide Association Studies data were utilized for this analysis. 33 single nucleotide polymorphisms known to be associated with GGT levels were employed as instrumental variables. We employed several MR methods including IVW (inverse variance weighting), MR-Egger, weighted median, weighted mode, and MR-PRESSO (Mendelian Randomization Pleiotropy RESidual Sum and Outlier). Furthermore, we conducted tests for horizontal multivariate validity, heterogeneity, and performed leave-one-out analysis to ensure the stability of the results. Overall, several MR methods yielded statistically significant results with a *p*-value < 0.05. The results from the IVW analysis yielded an odds ratio (OR) of 1.0062 with a 95% confidence interval (CI) of 1.0016–1.0109 (*p* = 0.0077). Additional MR methods provided supplementary results: MR-Egger (OR 1.0167, 95% CI 1.0070–1.0266, *p* = 0.0040); weighted median (OR 1.0058, 95% CI 1.0002–1.0115, *p* = 0.0423); and weighted mode (OR 1.0083, 95% CI 1.0020–1.0146, *p*- = 0.0188). Sensitivity analyses did not reveal heterogeneity or outliers. Although potential horizontal pleiotropy emerged, we speculate that this could be attributed to inadequate test efficacy. However, subsequent use of MR-PRESSO did not provide evidence of pleiotropy. Our analysis suggests a positive association between elevated GGT levels and CK incidence, indicating an increased risk of CK development. However, no causal relationship was observed between levels of ALP or ALT and CK incidence.

## Introduction

Urolithiasis is a common disease in urology. Individuals afflicted with urolithiasis frequently endure symptoms such as chronic pain, hematuria, urinary obstruction, and an increased risk of developing chronic conditions like renal insufficiency, all of which significantly affect their overall quality of life. The development of urolithiasis is influenced by a range of factors, encompassing age, gender, genetic predisposition, environmental influences, and dietary patterns. In addition to the mentioned factors, metabolic abnormalities, urinary tract obstruction, and drug use are common causes of stone formation^1^. Furthermore, unidentified factors continue to play a role in stone development. The direct and indirect costs of calculus of kidney (CK) will continue to rise in the United States, and efforts should be directed toward ameliorating the burden of urinary stone disease^2^. Consequently, it is imperative to understand the risk factors that contribute to CK formation. This knowledge is vital for prevention and cost reduction in treatment.

Most kidney stones consist of approximately 85% calcium combined with oxalate or phosphate. Notably, idiopathic hypercalciuria emerges as the primary risk factor for the formation of CK^3^. Gamma-glutamyl transferase (GGT) is involved in maintenance of physiological concentrations of glutathione (a primary antioxidant) in cells and reflects the oxidation-antioxidant balance in the body^4,5^. Furthermore, GGT serves as a key indicator of liver function. It is our expectation that insights into the development of CK can be gleaned from commonly utilized screening indicators, obviating the need for additional tests. This, in turn, will empower clinicians to assess the risk of CK development more effectively and refine treatment strategies, thereby mitigating the economic impact of stone management.

GGT exhibits high expression within the kidney and is believed to undergo shedding, releasing itself into both serum and urine. Purified GGT protein has the capacity to induce osteoclast formation, a process implicated in the release of calcium in bone pathology^6^, potentially contributing to idiopathic hypercalciuria. Vitro experiments have suggested a potential regulatory link between GGT and serum calcium levels^7^. Elevated levels of GGT typically indicate the onset of cholelithiasis. However, no reported association between GGT levels and other stone diseases, including kidney stones. Recent genetic analysis on individuals of European ancestry revealed the enrichment of GGT single nucleotide polymorphisms (SNPs) throughout various urogenital systems epithelial cells^8^. This observation has led us to speculate about a plausible link between GGT levels and CK incidence. To the best of our knowledge, such an association has not been established before. Consequently, we have applied Mendelian randomization (MR) analysis, in conjunction with publicly available medical statistical databases, to explore the potential relationship between GGT levels and CK incidence.

MR relies on the natural, random assortment of genetic variants during meiosis yielding a random distribution of genetic variants in a population^9^. This method aims to estimate causal effects by employing genetic variation as instrumental variables (IVs) to assess the causal relationship between exposures or risk factors and their subsequent outcomes. Typically, these IVs comprise one or more SNPs associated with the exposure of interest. These SNPs should demonstrate an association with the exposure while remaining unrelated to any confounding factors that might affect the association between the exposure and the outcome. Additionally, they should not affect outcomes through pathways other than the exposure^9–11^. MR analysis can be applied within the IV framework when these specific assumptions are met^12^. Leveraging publicly available Genome-Wide Association Studies (GWAS) databases and R-language code, researchers can efficiently explore causal links between exposure factors and disease outcomes in a cost-effective manner. This approach serves as a valuable tool for identifying new risk factors and charting innovative research directions.

In this study, we employed the TwoSampleMR package for MR analysis. We used published SNPs associated with GGT levels and summary statistics from related GWAS focusing on CK. Our primary goal was to ascertain a potential causal relationship between these variables. Furthermore, we expanded our study by incorporating SNPs associated with alanine transaminase (ALT) or alkaline phosphatase (ALP) levels. This supplementary analysis aimed to probe the potential influence of liver enzymes on CK incidence.

## Materials and methods

### Genetic variants associated with GGT levels

SNPs associated with GGT levels were used as IVs for MR analysis. These SNPs must satisfy three critical hypotheses^9^:

1. They are associated with GGT levels. (Association assumption)
2. They are not associated with confounding factors in GGT-CK relationship. (Independence assumption)
3. These SNPs exclusively influence stone pathogenesis through their impact on GGT levels. (Exclusive assumption)

We assessed association assumption by examining the strength of the association between GGT levels and genetic variants (with a significance threshold set at *p* < 1 × 10^−9^). Independence and exclusive assumptions (hypotheses 2 and 3) required validation through sensitivity analysis, as detailed in Section “Mendelian randomization”. These SNPs for MR analysis were sourced from a recent genome-wide study conducted by Pazoki et al.^8^ This study, involving 437,194 individuals of European ancestry, identified a total of 371 genomic loci associated with GGT levels. The raw data, including complete values for GGT concentration, were obtained from the UK-Biobank. Pazoki et al. applied specific criteria for SNP screening, which included: 1. Ensuring a c greater than 5% with a stringent significance level of *p* < 1 × 10^−8^ (compared to the usual GWAS threshold of *P* < 5 × 10^−8^, this stringent threshold was used to robustly define the lead SNPs for replication and functional assessment). 2. Pruning SNPs based on linkage disequilibrium, with a requirement that the r^2^ value be less than 0.1 within a 500-kilobase window. 3. Excluding specific categories of SNPs from the database, specifically: a. SNPs with multiple alleles. b. SNPs within the human leukocyte antigen (HLA) region, particularly those in the chromosomal range of chr6:25–34 MB. c. SNPs with a minor allele frequency (MAF) below 0.001. Then they amalgamated genetic loci previously identified by Chambers et al.^13^ and replicated the findings in three independent studies: the Rotterdam Study (NL, N = 6943), the Lifelines study (NL, N = 13,386), and the MVP (USA, N = 294,043). Ultimately, 200 SNPs associated with GGT levels were identified, comprising 167 previously undiscovered variants and 33 previously recognized. These SNPs satisfactorily meet the data requirements for MR analysis. For more extensive screening details, we encourage reference to the original article (https://doi.org/10.1038/s41467-021-22338-2).

To mitigate the risk of sample overlap, we selected 33 SNPs that had been previously identified for our MR analysis. Pazoki et al. compiled and publicly released the data for these 33 SNPs. Further details are available in Supplementary Table 1 (S_Table. 1).

### Genetic variants associated with ALP and ALT levels

Pazoki et al.^8^ also identified SNPs associated with levels of ALP and ALT, respectively. We applied the same filtering conditions and, for our analysis, utilized established SNPs rather than novel ones. For comprehensive details regarding the SNPs linked to ALP levels (a total of 25) and ALT levels (a total of 6), please consult Supplementary Tables 2 (S_Table. 2) and 3 (S_Table. 3).

### Summary data from GWAS on CK

The summary statistical data for CK were obtained from the UK-Biobank and are available on the IEU OPEN GWAS PROJECT (https://gwas.mrcieu.ac.uk)^14^. These GWAS results were based on samples of European ancestry, which included data from 2186 individuals diagnosed with CK and 460,824 control individuals, all of which are incorporated into the database.

### Mendelian randomization

We employed the TwoSampleMR (version 0.4.25) and MR-PRESSO (Mendelian Randomization Pleiotropy RESidual Sum and Outlier) packages (versions 1.0) in the R language software for data analysis.

After performing a genome-wide association analysis, we employed various MR methods to investigate the causal impact of GGT levels on CK. Our MR approaches included inverse variance weighting (IVW), weighted median, MR-Egger, and weighted mode. Each method considers distinct underlying assumptions and levels of pleiotropy, which collectively enhance the robustness of our estimates. The primary outcome was determined through an inverse variance-weighted meta-analysis of the Wald ratio for a single SNP. This analysis ensures that the instrumental variable solely influences the outcome through the target exposure, excluding alternative pathways. Furthermore, we confirmed the absence of correlations between instrumental variables. Under these assumptions, the IVW estimate represents the slope of the best fitting line through the data points which also passes through the origin. This approach is particularly suited for situations with strong genetic variation^15^. The MR-Egger analysis closely resembles IVW, but it takes the intercept term of the regression curve into account. The intercept in the MR-Egger analysis is assessed to account for pleiotropy within the analysis, making it a more suitable choice when the weaker assumption is met^16^. Additionally, we employed the MR-Egger, weighted median, and weighted mode methods to complement the IVW estimates, thus enhancing the overall robustness of the analysis^11^.

**Figure 1:**
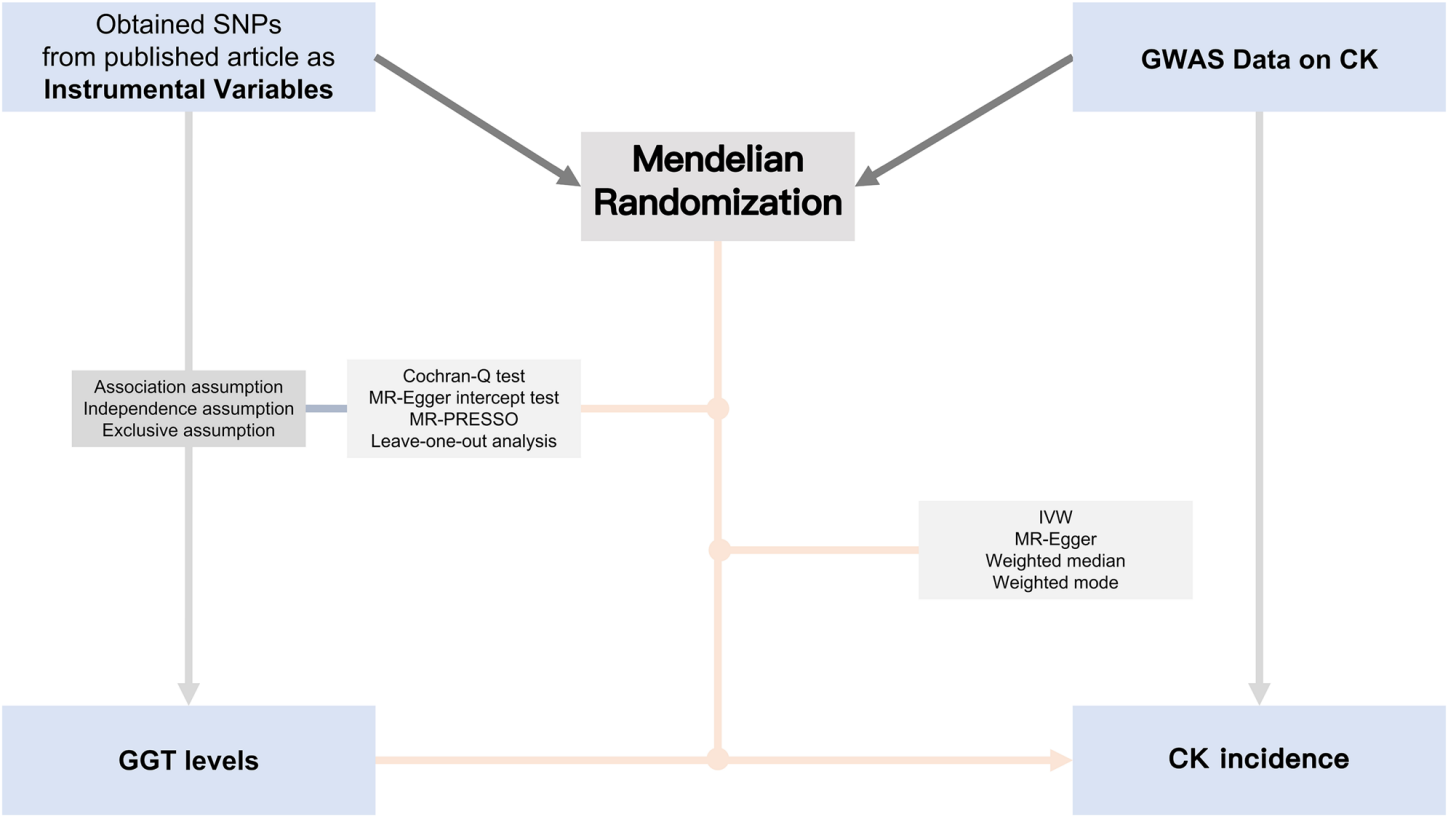
Workflow of Mendelian randomization study revealing causality from GGT levels on CK. SNP: single nucleotide polymorphism; GGT: Gamma-glutamyl transferase; CK: Calculus of kidney GWAS: Genome-Wide Association Studies; IVW: inverse variance weighting. MR-PRESSO: Mendelian Randomization Pleiotropy RESidual Sum and Outlier.

Sensitivity analysis plays a pivotal role in MR studies as it helps detect potential pleiotropy and heterogeneity, which can significantly affect MR estimates. These methods can be used to test hypotheses 2 and 3 as mentioned above^9^. Heterogeneity was evaluated using the Cochran-Q test. A *p*-value of ≤ 0.05, signifying the presence of pleiotropy, led us to employ the random effects Inverse Variance Weighting MR method^17^. Conversely, when the p-value exceeded 0.05 with no evidence of heterogeneity, we adopted the fixed effects IVW method as our primary approach^18^. To investigate horizontal pleiotropy, a statistical test was performed, setting the MR-Egger intercept term to 0. A *p*-value less than 0.05 suggested the presence of horizontal pleiotropy, indicating that the exposure factor may have influenced the outcome through other unknown factors, making reliable causal inference impossible^16^. Furthermore, we complemented the results of the horizontal pleiotropy test by utilizing MR-PRESSO analysis to identify any outliers in the data^19^. We also conducted leave-one-out analysis to evaluate whether MR estimates were driven or biased (as shown in Fig. 1).

Through the application of these MR methods, we successfully identified a causal relationship between GGT levels and CK incidence. Additionally, we conducted MR analyses of the other two liver enzymes, ALP and ALT levels, with CK to investigate their association. However, no causal relationship was found between these variables.

### Ethics approval and consent to participate

The authors are accountable for all aspects of the work in ensuring that questions related to the accuracy or integrity of any part of the work are appropriately investigated and resolved. The current analyses are based on publicly available summary data and therefore do not require ethical approval. Original studies have been approved by ethic committees and written informed consent was obtained from study participants or caregivers. The study was conducted in accordance with the Declaration of Helsinki (as revised in 2013).

## Results

### Causal relationship between GGT levels and CK

**Figure 2:**
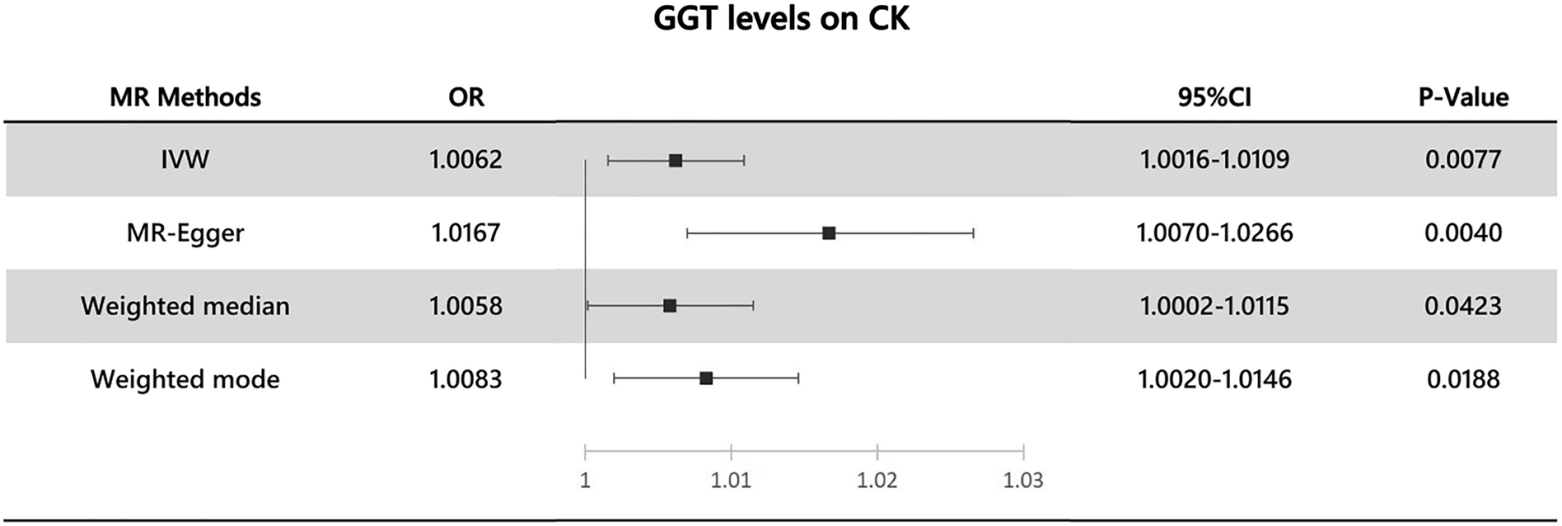
The association of GGT levels with CK outcomes by MR analysis through different methods: IVW (random effects), MR-Egger, Weighted median, Weighted mode. OR: odds ratio; CI: confidence interval.

Our analysis reveals significant statistical evidence of a causal relationship between elevated GGT levels and the incidence of CK. A random effects IVW MR analysis is appropriate, as indicated by the significant Cochran's Q test (*p* < 0.05), which suggests the presence of potential heterogeneity. The IVW analysis yielded an odds ratio (OR) of 1.0062, with a 95% confidence interval (CI) from 1.0016 to 1.0109 and a p-value of 0.0077, indicating an association between elevated GGT levels and an increased risk of CK incidence. This finding was supported by additional MR methods: MR-Egger, weighted median and weighted mode method corroborated these findings: MR-Egger: OR of 1.0167, 95% CI (1.0070–1.0266), and *p*-value of 0.0040. Weighted Median: OR of 1.0058, 95% CI (1.0002–1.0115), and *p*-value of 0.0423. Weighted Mode: OR of 1.0083, 95% CI (1.0020–1.0146), with a *p*-value of 0.0188 (as shown in Fig. 2).

**Figure 3:**
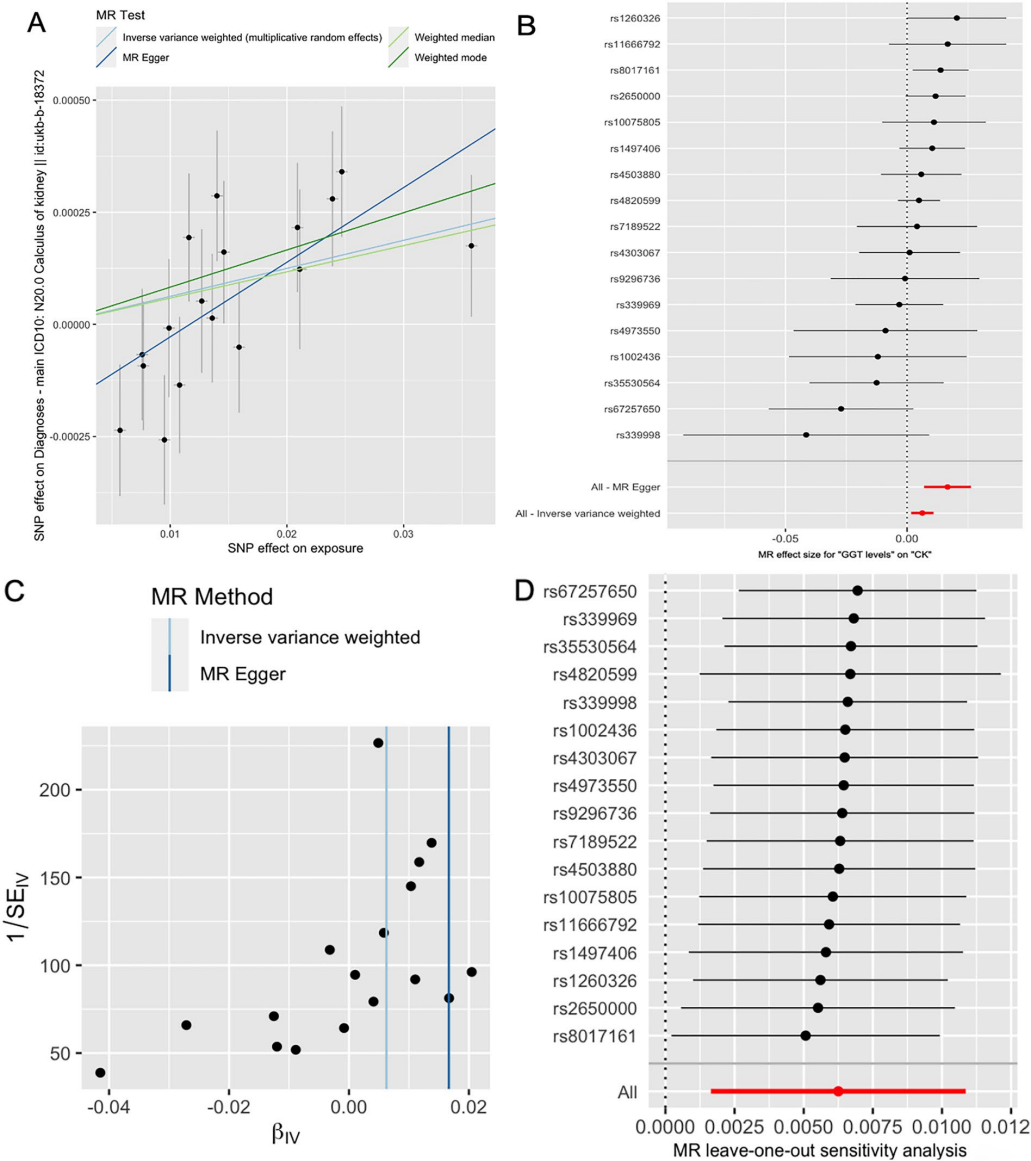
Association between GGT levels and CK incidence. (**A**) multiple MR tests showed the SNP effects; (**B**) effect size of each SNPs; (**C**) funnel plot for GGT levels of CK incidence; (**D**) leave-one-out sensitivity analysis. CK: calculus of kidney; MR: Mendelian Randomization.

Notably, we observed an intercept (intercept = − 0.001, se = 0.00008, *p* = 0.0320), which cloud suggest the presence of horizontal pleiotropy. However, it's essential to consider that this observation may be influenced by limitations in the MR-Egger method's testing efficacy^16^. To further investigate this situation, we conducted MR-PRESSO analysis. The global test results revealed no significant evidence of horizontal pleiotropy (*p* = 0.3264). Furthermore, the symmetry of the funnel plot provides additional support for these findings. Both the outlier test and the leave-one-out analysis failed to identify any potentially influential SNPs that could introduce bias into the causal associations. Consequently, these outcomes reinforce the robustness of our conclusions. Verification through both outlier tests and leave-one-out analysis confirmed that the SNPs under study did not exhibit any potential effects that could introduce bias into the causal associations. Therefore, these finding provide strong support for the stability and reliability of our conclusions (as shown in Fig. 3A–D).

### Causal relationship between ALT or ALP and CK

We employed the same methods to investigate the relationship between ALT or ALP levels and CK incidence. Both ALT and ALP are conventional indicators of liver function. However, after excluding incompatible alleles and SNPs with homozygotes at intermediate allele frequencies. 25 SNPs associated with ALP levels were involved in the MR analysis. The IVW analysis indicated a possible causal link between ALP levels and CK incidence (*p* < 0.05), but this result lacked sufficient test efficacy for sensitivity analysis. The same analysis was used for the relationship between ALT levels and CK onset. However, there was no evidence of a causal relationship between the two. In summary, among the three liver enzymes associated with liver function, only GGT levels were causally linked to CK onset.

## Discussion

Urolithiasis is a prevalent disease in urology that can significantly impact a patient's quality of life during an episode. Despite our general understanding of the causes and risk factors associated with urolithiasis, its prevention remains challenging. The diagnosis of CK relies on imaging and the patient's report of painful symptoms^20^. The formation of stones is a multifaceted process. While supersaturation, a key driver of calcium stone formation, plays a central role, it is influenced by stone type, comorbidities, and urine chemistry^3^. There exists a positive correlation between metabolic acidosis or endogenous acid production and increased urinary calcium excretion^21^. Increased calcium release from bone is an important contributor to excessive calcium excretion in urine in idiopathic hypercalciuria^3^. The buffering effect of protons promotes bone loss through physicochemical mineral lysis (acute) and cell-mediated bone resorption (chronic), both of which are achieved through the activation of osteoclasts and osteoblasts^22^. During the buffering process, a significant amount of cations, primarily calcium, enters the circulation and is excreted by the kidneys^21^. This reflects alterations in tubular calcium handling and is a critical factor contributing to the formation of calcium-containing kidney stones^3^. GGT serves as a cytokine involved in osteoblast development and does not require enzymatic activity^6^. It triggers expression of the receptor activator of nuclear factor-kappa B ligand (RANKL), leading to the differentiation of osteoblasts into osteoclasts^23^. We hypothesize that the induction process on osteoclasts could be a potential mechanism linking elevated GGT levels to CK formation. Inflammation, oxidative-antioxidant imbalances, purine metabolism, and the urea cycle all play vital roles in the dynamics of urinary tract deposits. CaOX crystal deposition may cause mitochondrial damage through increased ceramide levels, a process whose results include glutathione depletion, which leads to activation of cysteinyl asparaginase, induces apoptosis, and accelerates the development of renal stones^24^. The regulatory function of GGT on glutathione metabolism may elucidate the potential mechanism underlying the connection between GGT and CK pathogenesis. Recent studies have revealed relationships between inflammatory vesicles, caspase-1, and apoptosis^25^, which could further aid in understanding the underlying regulatory mechanisms.

Bioinformatics methods offer valuable insights for prediction and mechanism exploration^26^. Combining Mendelian randomization with machine learning can facilitate predictive research. The graph convolutional network with graph attention network (GCNAT) deep learning algorithm can facilitate predictive research. The GCNAT algorithm, for instance, predicts metabolite-disease associations^27^, which can inform future studies on GGT levels and CK pathogenesis. Recent research has introduced novel models for the phase separation of messenger RNAs. The theoretical framework of intracellular phase separation, grounded in this specific physical property, holds promise in facilitating drug target discovery and advancing disease prevention^28^. Non-coding RNAs also play roles in formation of CK. Rat gene sequencing revealed differential expression of Long non-coding RNA (lncRNA) and Circular RNA (circRNA) affecting kidney stone occurrence^29^. Mathematical models, including graph-based convolutional neural networks (GCN) and conditional random fields (CRF), predict human lncRNA-miRNA interactions^30^. The MPCLCDA model effectively predicts circRNA–disease associations by using automatically selected meta-path and contrastive learning^31^, while the network structure refinement method for gene regulatory networks (NSRGRN) model optimally elucidates gene regulatory networks^32^. However, it's important to acknowledge the limitations of this study:

1. Our GWAS data were derived solely from European populations, excluding other ethnic groups. Even within the same ethnic group, results may vary between different databases. Therefore, the generalizability of our results is limited to the specific databases and populations we analyzed.
2. The MR methods we employed, while powerful, may not eliminate potential biases completely. Our results explain the causal relationship between the two variables using statistical methods. Further research, including mathematical modeling and randomized controlled trials (RCTs), is needed to validate and support these findings.

## Conclusion

This is the first study to link GGT levels to the risk of kidney stones using MR analysis. Our analysis suggests a positive association between elevated GGT levels and CK incidence, indicating an increased risk of CK development, while no causal relationship was observed between levels of ALP or ALT and CK incidence.

### Supplementary Information

Supplementary Table 1.

Supplementary Table 2.

Supplementary Table 3.

## Data Availability

Publicly available datasets were utilized for the analysis in this study. Specifically, the levels of GGT, ALT, and ALP were obtained from a recent genome-wide study conducted by Pazoki et al. (https://doi.org/10.1038/s41467-021-22338-2). The summary statistical data on CK can be accessed through the IEU OPEN GWAS PROJECT (https://gwas.mrcieu.ac.uk), with the GWAS ID being ukb-b-18372.

## Abbreviations

GGT: Gamma-glutamyl transferase
SNP: Single nucleotide polymorphism
CK: Calculus of kidney
MR: Mendelian randomization
ALT: Alanine transaminase
ALP: Alkaline phosphatase
IVW: Inverse variance weighting
MR-PRESSO: Mendelian Randomization Pleiotropy RESidual Sum and Outlier
OR: Odds ratio
CI: Confidence interval
GWAS: Genome-Wide Association Studies
HLA: Human leukocyte antigen
MAF: Minor allele frequency
RANKL: Receptor activator of nuclear factor-kappa B ligand
GCNAT: Graph convolutional network with graph attention network
GCN: Graph-based convolutional neural networks
CRF: Conditional random fields
NSRGRN: Network structure refinement method for gene regulatory networks
RCT: Randomized controlled trial
